# The cross-sectional association between cardiometabolic index and abdominal aortic calcification in U.S. adults: evidence from NHANES 2013–2014

**DOI:** 10.3389/fnut.2025.1537795

**Published:** 2025-07-02

**Authors:** Jiawei Peng, Jijun Wu, Xitu Luo, Chengyu Yang, Shian Wu, Wenjun Liu, Yuanhao Feng

**Affiliations:** ^1^Department of Vascular Surgery, Guangdong Provincial Key Laboratory of Major Obstetric Diseases, Guangdong Provincial Clinical Research Center for Obstetrics and Gynecology, The Third Affiliated Hospital, Guangzhou Medical University, Guangzhou, China; ^2^Department of Interventional Radiology, Zhongshan Torch Development Zone People’s Hospital, Zhongshan, China; ^3^Department of General Surgery, The Sihui People’s Hospital, Zhaoqing, China; ^4^The First School of Clinical Medicine, Southern Medical University, Guangzhou, China

**Keywords:** cardiometabolic index, abdominal aortic calcification, NHANES, metabolism, cross-sectional association

## Abstract

**Background:**

The cardiometabolic index (CMI) is a novel composite measure that integrates assessments of abdominal adiposity and lipid profiles. While abdominal aortic calcification (AAC) is a well-established marker of subclinical atherosclerosis and systemic metabolic dysregulation, the association between CMI and AAC remains underexplored. This cross-sectional study aimed to investigate the association between CMI and AAC.

**Methods:**

A cross-sectional study was conducted using data from the 2013 to 2014 National Health and Nutrition Examination Survey (NHANES) to explore the relationship between CMI and AAC. A weighted multivariate logistic regression model was employed to assess the associations between triglycerides (TG), high-density lipoprotein cholesterol (HDL-C), waist-to-height ratio (WHtR), CMI, and AAC. The area under the receiver operating characteristic (ROC) curve (AUC) was used to assess the statistical association strength of each variable with AAC presence. Non-linear relationships were examined through restricted cubic spline (RCS) curve analysis. Potential influencing factors were investigated through subgroup analysis.

**Results:**

The average CMI of 2,675 participants was 0.98 ± 1.36. Multivariable regression showed that each one-unit increase in lnCMI was associated with a 0.19-point increase in the AAC score (β = 0.19, 95% CI: 0.03–0.35). Individuals in the highest CMI group had a 34% higher likelihood of severe AAC than those in the lowest (OR = 1.34; 95% CI, 1.09–1.66, *P* < 0.05). The ROC analysis showed CMI had an AUC of 0.548, comparable to TG (0.545), HDL-C (0.526), and WHtR (0.525). Although differences were not statistically significant (all *P* > 0.05), CMI may reflect underlying metabolic characteristics associated with AAC. A significant trend (*P* < 0.05) indicated a non-linear CMI-AAC relationship with gender-based interactions.

**Conclusion:**

This study demonstrated a positive correlation between CMI and AAC. However, given the cross-sectional nature of the study, causality cannot be directly inferred. These cross-sectional findings indicate a statistical association between CMI and AAC burden, suggesting potential epidemiological relevance. However, no causal inference can be drawn, but further longitudinal cohort studies are needed to confirm its potential value.

## Introduction

The global prevalence of cardiovascular disease (CVD) continues to rise, driven by an aging population and changing lifestyle factors, making it the leading cause of death worldwide. This poses significant challenges for individuals and healthcare systems alike (1). Recent studies have highlighted the growing age and socioeconomic disparities in CVD incidence, with these disparities showing persistence over time (2). Abdominal aortic calcification (AAC), a common form of vascular calcification (VC), is a key independent risk factor for CVD (3).

VC, characterized by the accumulation of mineral deposits, particularly calcium phosphate complexes, within the blood vessels (4), contributes significantly to vascular stiffness, especially in the aorta. Increased aortic stiffness is an established independent risk factor for cardiovascular diseases (5). In older adults, the progression of aortic stiffness is also strongly linked to poorer socioeconomic status (6). Beyond serving as a reliable indicator of atherosclerotic disease, AAC has been associated with increased cardiovascular events and all-cause mortality in prior studies (7, 8). Calcified plaques in the aorta are associated with structural changes, including increased aortic diameter, which can lead to the rupture of aortic aneurysms (9, 10). Furthermore, the presence of extensive calcification complicates and heightens the risks of open surgical procedures (11). Although abdominal aortic aneurysm is less prevalent in women, their prognosis tends to be worse, likely due to less effective cardiovascular risk management in females (12, 13). Population-based studies have indicated a correlation between the severity of AAC and spinal X-ray findings (14).

Recent attention has focused on the relationship between metabolic status and AAC (15, 16). Individuals with aortic calcification tend to be older, have elevated cholesterol levels, and are more likely to have diabetes than those without calcification (17, 18). Obesity has been identified as a key factor that increases the likelihood of severe AAC (19). In obese individuals, altered lipid metabolism and systemic metabolic disruptions contribute to this increased risk, positioning lipid profiles as valuable biomarkers for AAC (20). Interestingly, men with lower income levels show higher rates of obesity, while wealthier women tend to have a higher prevalence of obesity as well. The reasons for racial disparities in lipid management remain poorly understood (21, 22), underscoring the importance of assessing body fat accumulation as a risk indicator for AAC, with consideration of factors such as gender, race, and socioeconomic status (23). In clinical settings, the diagnosis of lipid metabolism disorders typically requires a combination of anthropometric data and biochemical markers. The cardiometabolic index (CMI), which includes metrics such as the waist-to-height ratio (WHtR) and the triglyceride (TG)/high-density lipoprotein cholesterol (HDL-C) ratio, has been associated with utility in identifying individuals with diabetes and obesity (24, 25). Numerous studies have reported associations between CMI and various conditions including fatty liver disease, hypertension, atherosclerosis, chronic kidney disease (CKD), and depression (26–28). CMI has been proposed as a potential tool for assessing cardiometabolic risk at the population level. When compared to individual risk factors, the CMI offers superior predictive value, providing a holistic assessment of an individual’s cardiac metabolic risk. This makes it an essential reference for early prediction, evaluation, and clinical decision-making in cardiovascular health.

Compared with indices like LAP and VAI, CMI combines the TG/HDL-C ratio and waist-to-height ratio (WHtR), capturing both dyslipidemia and central adiposity. These components are key drivers of metabolic syndrome and vascular damage, particularly relevant to AAC pathogenesis (27). Currently, there is a lack of literature exploring the association between the cardiometabolic index (CMI) and abdominal aortic calcification (AAC). A better understanding of this relationship may offer insights into the cardiometabolic characteristics associated with AAC. This study, based on cross-sectional data from the 2013 to 2014 NHANES cycle, aims to evaluate the statistical association between CMI and both the prevalence and severity of AAC, while exploring potential contributing factors behind this association.

## Materials and methods

### Study design and study population

This study utilized a cross-sectional analysis based on NHANES data. The NHANES survey is a comprehensive effort to collect health-related information from the U.S. population (29). To ensure the national sample’s representativeness, a stratified multistage random sampling method was employed. Ethical approval for NHANES was granted by the National Center for Health Statistics, and informed consent was obtained from all participants (30).

**FIGURE 1:**
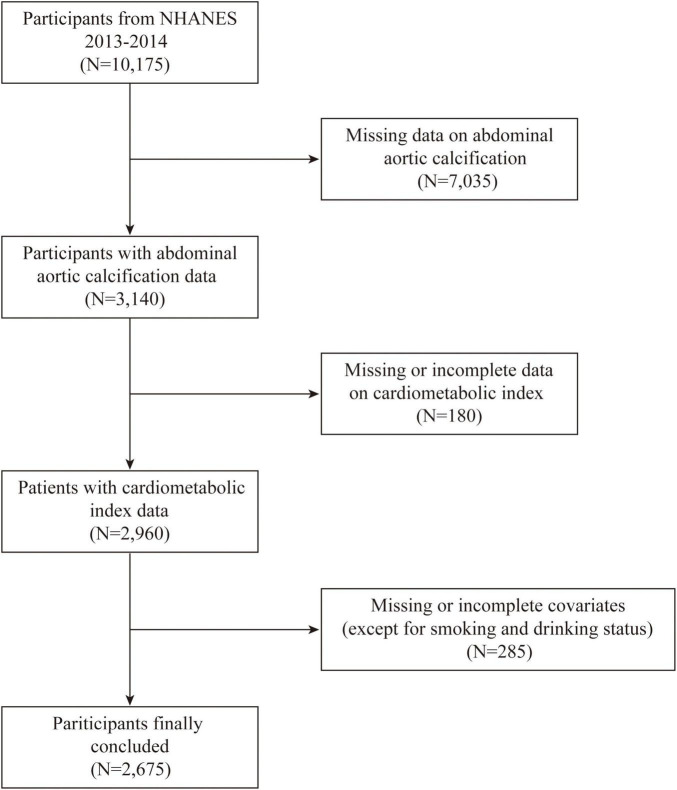
Flowchart showing the inclusion and exclusion of the study population.

Only the 2013–2014 NHANES cycle was used in this analysis, as it is the only cycle containing data on AAC, resulting in a final sample of 2,675 individuals. The dataset included detailed demographic information, standardized anthropometric measurements, lipid profiles, and comprehensive health status data. The inclusion and exclusion criteria are illustrated in Figure 1. The exclusion criteria were as follows: (1) missing data on abdominal aortic calcification, (2) missing or incomplete CMI data, (3) missing or incomplete covariate data, (4) pregnant participants, and (5) individuals under 18 years of age.

### Assessment of CMI

CMI was calculated using the subject’s waist circumference-to-height ratio and lipid profile. The formula used was: CMI = (TG/HDL-C) × (waist circumference/height). The natural logarithm of CMI was also computed: lnCMI = ln(CMI) (24).

### Assessment of AAC

In this study, dual-energy X-ray absorptiometry (DXA) was utilized to assess AAC (31, 32). The AAC score, ranging from 0 to 24, measured the severity of calcification in the abdominal aorta. DXA was performed on individuals aged ≥ 40 years, with exclusion criteria including (1) pregnancy, (2) radiological procedures involving barium contrast within the previous week, (3) body weight exceeding 450 pounds, or (4) the presence of a Harrington rod for scoliosis correction. Severe AAC, defined as a Kauppila score > 6, was treated as a binary outcome (33).

### Covariates

Data on several covariates were collected for this study, including age, sex, race, education level, smoking status, alcohol consumption, body mass index (BMI), poverty-income ratio (PIR), serum calcium, serum phosphate, total 25-hydroxyvitamin D (nmol/L), uric acid, blood urea nitrogen, TGs, HDL-C, WHtR, diabetes, hypertension, chronic kidney disease, CVD, asthma, arthritis, chronic obstructive pulmonary disease (COPD), and cancer. Poverty was defined as a household income-to-poverty ratio ≤ 1.3 (34). The criteria for chronic diseases, smoking, and alcohol consumption were based on NHANES standards. Smoking and alcohol consumption were defined according to the New Zealand Ministry of Health guidelines (35). Diabetes was defined based on the following criteria: (1) fasting blood glucose ≥ 7.0 mmol/L; (2) blood glucose level during the oral glucose tolerance test > 11.1 mmol/L; (3) random blood glucose level > 11.1 mmol/L; (4) glycated hemoglobin > 6.5%; (5) use of diabetes medications; or (6) self-reported diabetes diagnosis (36). Hypertension was defined as: (1) self-reported diagnosis of “high blood pressure,” “having high blood pressure twice,” or “taking prescription medication for high blood pressure”; and (2) average blood pressure measurements from NHANES of ≥ 130 mmHg systolic or ≥ 80 mmHg diastolic based on three measurements (37). CKD was characterized by a urine albumin-to-creatinine ratio ≥ 30 mg/g or 3 mg/mmol, alongside an estimated glomerular filtration rate < 60 mL/min/1.73 m^2^ (38). CVD was defined based on self-reports of stroke, angina, myocardial infarction, coronary heart disease, or heart failure (39). Asthma was defined as self-reported diagnosis of “being told they have asthma” or “age at which they were diagnosed with asthma” between the age of 1 and 80 years (40). Arthritis was characterized as self-reported instances of “being informed they have arthritis” or “age at which they were informed of having arthritis” between 1 and 80 years, or specifying the type of arthritis. COPD was identified by self-reported diagnosis of chronic obstructive lung disease (41). Cancer was defined as self-reported “diagnosis of cancer” or “age at which they were first diagnosed with cancer” between 1 and 80 years, or specifying the type of cancer (40).

### Statistical analysis

All statistical analyses applied sample weights to ensure that the estimated data accurately represented the national demographic structure. Analyses were performed using R software (version 4.2.2). For continuous variables, means and standard deviations were used, while percentages were applied to categorical variables. Baseline continuous and categorical variables were analyzed using analysis of variance and weighted chi-square tests, respectively. To examine the relationship between CMI and AAC, three distinct multiple logistic regression models were employed: Model 1 (unadjusted), Model 2 (adjusted for age and race), and Model 3 (adjusted for all covariates). The same method, including covariate adjustment, was used to calculate β (95% CI) and odds ratios (ORs, 95% CI) for AAC, as well as for TG, HDL-C, and WHtR. The association strength of CMI and related indicators with severe AAC was evaluated via ROC curves and corresponding AUCs. Statistical comparisons between area under the curve (AUC) values were performed using the Z test. Additionally, restricted cubic spline (RCS) curves derived from Model 3 were employed to investigate potential non-linear associations between the CMI index and AAC. Baseline characteristics across different CMI quartiles were compared using chi-square tests and Kruskal-Wallis H tests to assess differences in age, sex, race, BMI, and other variables across CMI groups. Subgroup analyses were performed to evaluate the influence of age, sex, and race on the association between CMI and AAC using interaction terms. Statistical significance was set at *P* < 0.05.

## Results

### Characteristics of participants

According to the established inclusion and exclusion criteria, 2,675 adults participated in this study. The average age was 58.62 ± 12.01 years, with 12.49% identified as Mexican American, 45.64% as non-Hispanic White, 19.03% as non-Hispanic Black, and 22.84% from other ethnic backgrounds. Participants with higher CMI levels were more likely to be non-Hispanic Black, non-Hispanic White, and male. The mean BMI and waist circumference for the group were 28.50 ± 5.61 kg/m^2^ and 99.43 ± 13.78 cm, respectively. Table 1 presents the baseline characteristics of participants stratified by CMI quartiles. The final study population had an average CMI of 0.98 ± 1.36. The quartiles for CMI were defined as follows: CMI_Q1 (≤ 0.36), Q2 (0.36–0.64), Q3 (0.64–1.20), Q4 (> 1.20). The findings revealed significant variations in demographic and clinical characteristics across groups with differing CMI levels. Among the quartiles, notable differences were observed in the distribution of race, education level, BMI, PIR, diabetes, hypertension, CKD, total 25-hydroxyvitamin D levels, serum uric acid, TG, HDL-C levels, and WHtR (all *p* < 0.05). Individuals in higher CMI quartiles exhibited larger waist measurements, higher BMI, and a greater incidence of diabetes, hypertension, hyperuricemia, and CKD compared to those in the lowest CMI group. Additionally, compared to the other quartiles, those in quartile 4 showed elevated levels of TG and WHtR, while exhibiting lower levels of HDL-C.

**TABLE 1:** Weighted baseline characteristics of study participants according to quartile groups of cardiometabolic index.

Characteristics	Overall	Cardiometabolic index				*P*-value
	*n* = 2675	Q1 (*N* = 669)	Q2 (*N* = 669)	Q3 (*N* = 668)	Q4 (*N* = 669)	
**Demographics**						
Age (years)	58.62 ± 12.01	58.00 ± 12.41	58.93 ± 12.02	59.51 ± 11.97	57.97 ± 11.57	0.375
Gender (%)						< 0.001
Male	1,298(48.52)	266(39.76)	309(46.19)	330(49.40)	393(58.74)	
Female	1377(51.48)	403 (60.24)	360(53.81)	338(50.60)	276(41.36)	
Race, n (%)						< 0.001
Mexican American	334 (12.49)	41 (6.13)	79 (11.81)	95 (14.22)	119 (17.79)	
Non-Hispanic Black	509 (19.03)	194 (29.00)	147 (21.97)	114 (17.07)	54 (8.07)	
Non-Hispanic White	1221 (45.64)	298 (44.54)	290 (43.35)	304 (45.51)	329 (49.18)	
Others	611 (22.84)	136 (20.33)	153 (22.87)	155 (23.20)	167 (24.96)	
Education level, n (%)						0.007
< High school	577 (21.57)	121 (18.09)	135 (20.18)	149 (22.31)	172 (25.71)	
> High school	1492 (55.76)	414 (61.88)	387 (57.85)	369 (55.24)	322 (48.13)	
High school	606 (22.65)	134 (20.03)	147 (21.97)	150 (22.46)	175 (26.16)	
BMI (kg/m^2^), n (%)						< 0.001
<25	758 (28.34)	358 (53.51)	207 (30.94)	125 (18.71)	68 (10.16)	
≥ 30	953 (35.63)	105 (15.70)	200 (29.90)	301 (45.06)	347 (51.87)	
25 to < 30	964 (36.04)	206 (30.79)	262 (39.16)	242 (36.23)	254 (37.97)	
PIR, n (%)						0.002
< 1.3	794 (29.68)	165 (24.66)	181 (27.06)	211 (31.59)	237 (35.43)	
≥ 3.5	950 (35.51)	279 (41.70)	250 (37.37)	227 (33.98)	194 (29.00)	
1.3 to < 3.5	931 (34.80)	225 (33.63)	238 (35.58)	230 (34.43)	238 (35.58)	
**Lifestyle factors**						
Drinking alcohol, n (%)						0.139
Heavy drinker	244 (9.12)	82 (12.26)	61 (9.12)	52 (7.78)	49 (7.32)	
Low to moderate drinker	1694 (63.33)	415 (62.03)	430 (64.28)	410 (61.38)	439 (65.62)	
Non-drinker	737 (27.55)	172 (25.71)	178 (26.61)	206 (30.84)	181 (27.06)	
Smoke, n (%)						0.299
Current	499 (18.65)	123 (18.39)	120 (17.94)	120 (17.96)	136 (20.33)	
Former	757 (28.30)	164 (24.51)	189 (28.25)	202 (30.24)	202 (30.19)	
Never	1419 (53.05)	382 (57.10)	360 (53.81)	346 (51.80)	331 (49.48)	
**Comorbidities**						
Diabetes mellitus, n (%)	551 (20.60)	67 (10.01)	118 (17.64)	157 (23.50)	209 (31.24)	< 0.001
Hypertension, n (%)	1737 (64.93)	388 (58.00)	415 (62.03)	465 (69.61)	469 (70.10)	< 0.001
CKD, n (%)	585 (21.87)	122 (18.24)	122 (18.24)	169 (25.30)	172 (25.71)	0.021
CVD, n (%)	348 (13.01)	71 (10.61)	83 (12.41)	90 (13.47)	104 (15.55)	0.131
Asthma, n (%)	371 (13.87)	96 (14.35)	93 (13.90)	87 (13.02)	95 (14.20)	0.545
Arthritis, n (%)	918 (34.32)	204 (30.49)	232 (34.68)	235 (35.18)	247 (36.92)	0.113
COPD, n (%)	116 (4.34)	25 (3.74)	29 (4.33)	26 (3.89)	36 (5.38)	0.493
Cancer, n (%)	345 (12.90)	90 (13.45)	85 (12.71)	88 (13.17)	82 (12.26)	0.77
**Biochemical indicators**						
Serum calcium (mmol/L)	2.36 ± 0.09	2.36 ± 0.09	2.36 ± 0.09	2.36 ± 0.09	2.37 ± 0.10	0.315
Serum phosphorus (mmol/L)	1.22 ± 0.18	1.23 ± 0.17	1.22 ± 0.17	1.22 ± 0.19	1.23 ± 0.20	0.477
Total 25-hydroxyvitamin D (nmol/L)	70.56 ± 29.52	72.94 ± 31.76	71.13 ± 29.58	71.54 ± 29.83	66.64 ± 26.35	< 0.001
Serum uric acid (μmol/L)	324.06 ± 82.19	295.45 ± 78.11	317.75 ± 75.64	329.86 ± 80.47	353.20 ± 83.73	< 0.001
Blood urea nitrogen (mg/dl)	5.10 ± 2.20	4.85 ± 1.95	5.04 ± 2.08	5.22 ± 2.45	5.29 ± 2.28	0.273
TG (mmol/L)	1.81 + 1.82	0.76 ± 0.22	1.21 ± 0.29	1.78 ± 0.45	3.49 ± 2.93	< 0.001
HDL-C (mmol/L)	1.40 + 0.43	1.85 ± 0.45	1.46 ± 0.28	1.26 ± 0.24	1.02 ± 0.21	< 0.001
WHtR	0.60 + 0.08	0.54 ± 0.07	0.59 ± 0.08	0.62 ± 0.07	0.64 ± 0.07	< 0.001

Mean ± standard deviation for continuous variables, the *P*-value was calculated by the weighted linear regression model; n (%) for categorical variables, the *P*-value was calculated by the weighted chi-square test. PIR, poverty income ratio; CKD, chronic kidney disease; CVD, cardiovascular disease; COPD, chronic obstructive pulmonary disease; TG, triglyceride; HDL-C, high-density lipoprotein cholesterol; WHtR, waist-to-height ratio.

### Associations of CMI with AAC

The results suggest that higher CMI is associated with an elevated AAC score and a greater likelihood of severe AAC (Table 2). In the unadjusted model (Model 1), each unit increase in lnCMI was associated with a 0.21-point higher AAC score and a 27% greater odds of severe AAC. Among the CMI quartiles, Q1 served as the reference group, while Q2–Q4 exhibited varying associations with AAC scores and severe AAC in different models. In the minimally adjusted model (Model 2), lnCMI remained positively associated with both AAC score (β = 0.17) and severe AAC (OR = 1.33). In adjusted model 3, the associations between lnCMI and AAC scores, as well as severe AAC, remained significant, though the coefficients varied. The β value for HDL-C and AAC score was –0.43 (95% CI: –0.75 to –0.11, *P* = 0.008), indicating a negative correlation. The OR for TG and severe AAC was 1.07 (95% CI: 1.02–1.12, *P* = 0.014), showing a positive correlation. The OR for HDL-C and severe AAC was 0.58 (95% CI: 0.37–0.91, *P* = 0.031), reflecting a negative correlation. HDL-C showed a negative association with AAC scores, whereas TG exhibited a positive trend with AAC scores in the crude model (Supplementary Table S1).

**TABLE 2:** Associations between CMI and AAC.

CMI	AAC score		Severe AAC	
	**β (95% CI)**	***p*-value**	**OR (95% CI)**	***p*-value**
**Crude model (Model 1)**				
lnCMI	0.21 (0.05−0.36)	0.008	1.27 (1.06−1.53)	0.012
**Categories**				
Q1	0 (ref)		1 (ref)	
Q2	0.25 (−0.12 to 0.62)	0.187	1.67 (0.92–3.03)	0.087
Q3	0.39 (0.02–0.77)	0.039	1.69 (0.91–3.14)	0.087
Q4	0.54 (0.17–0.91)	0.005	1.98 (1.18–3.34)	0.014
P for tend	0.18 (0.06–0.29)	0.003	1.21 (1.05–1.40)	0.013
**Minimally adjusted model (Model 2)**				
lnCMI	0.17 (0.03–0.31)	0.020	1.33 (1.06–1.66)	0.028
**Categories**				
Q1	0 (ref)		1 (ref)	
Q2	0.14 (−0.20 to 0.48)	0.421	1.56 (0.86–2.85)	0.165
Q3	0.20 (−0.15 to 0.54)	0.268	1.50 (0.80–2.80)	0.226
Q4	0.46 (0.11–0.81)	0.011	2.12 (1.12–4.03)	0.036
P for tend	0.14 (0.03–0.25)	0.012	1.24 (1.03–1.50)	0.039
**Fully adjusted model (Model 3)**				
lnCMI	0.19 (0.03–0.35)	0.021	1.34 (1.09–1.66)	0.014
**Categories**				
Q1	0 (ref)		1 (ref)	
Q2	0.22 (−0.12 to 0.57)	0.202	1.78 (0.98–3.23)	0.078
Q3	0.30 (−0.07 to 0.66)	0.109	1.73 (0.97–3.08)	0.081
Q4	0.51 (0.13–0.90)	0.010	2.19 (1.26–3.82)	0.015
P for tend	0.16 (0.04–0.28)	0.011	1.25 (1.06–1.47)	0.019

Model 1: No adjustment for covariates; Model 2: Adjusted for age, sex, race, and education level; Model 3: Adjusted for smoking, alcohol use, BMI, PIR, DM, hypertension, CKD, CVD, asthma, arthritis, COPD, cancer, serum calcium, serum phosphorus, total 25-Hydroxyvitamin D, uric acid, and blood urea nitrogen based on Model 2.

**FIGURE 2:**
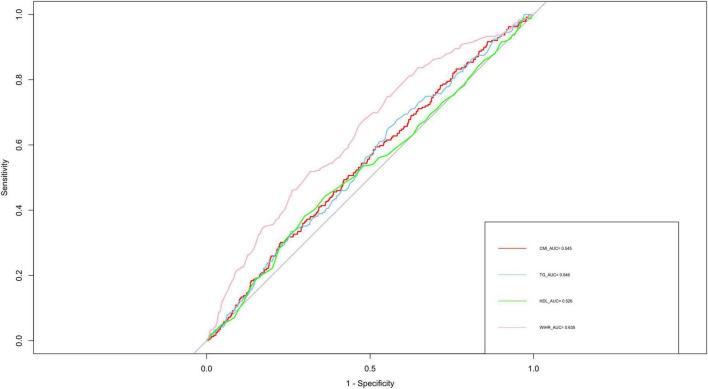
ROC curve analysis of various obesity indicators for predicting periodontitis. ROC, receiver operating characteristic; AUC, area under the curve; TG, triglyceride; HDL-C, high-density lipoprotein cholesterol; WHtR, waist-to-height ratio; CMI, cardiometabolic index.

**FIGURE 3:**
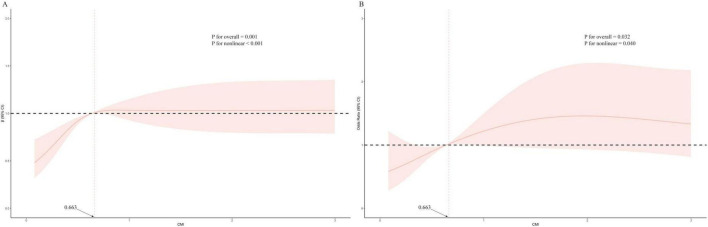
Restricted cubic spline plots of relationships between CMI score with AAC score **(A)**, and the risk of severe AAC **(B)** in the study population. Multivariable adjusted β value for AAC score and odds ratio (OR) for the risk of severe AAC in model 3. Statistically significant non-linear relationships were observed in both panels (P for non-linearity < 0.05). In panel B, a visible inflection point is present at CMI = 0.663, indicating a range in which the statistically positive association with severe AAC becomes more pronounced. CMI, cardiometabolic index; AAC, abdominal aortic calcification.

The ROC analysis revealed that CMI had an AUC of 0.548, which was similar to TG (0.545), HDL-C (0.526), and WHtR (0.525) (Figure 2). Although these AUC values reflect modest statistical association, CMI is not intended as a standalone diagnostic tool. Rather, it may reflect underlying metabolic conditions associated with AAC. To further characterize the association between CMI and AAC, we conducted restricted cubic spline (RCS) analysis (Figure 3), which demonstrated a non-linear association between CMI and the odds of severe AAC. A notable inflection point was observed at CMI ≈ 0.66, beyond which a stronger positive association with severe AAC was evident. Given the cross-sectional nature of the NHANES data, this finding should be interpreted as a statistical correlation rather than a causal or predictive relationship.

### Subgroup analysis

**FIGURE 4:**
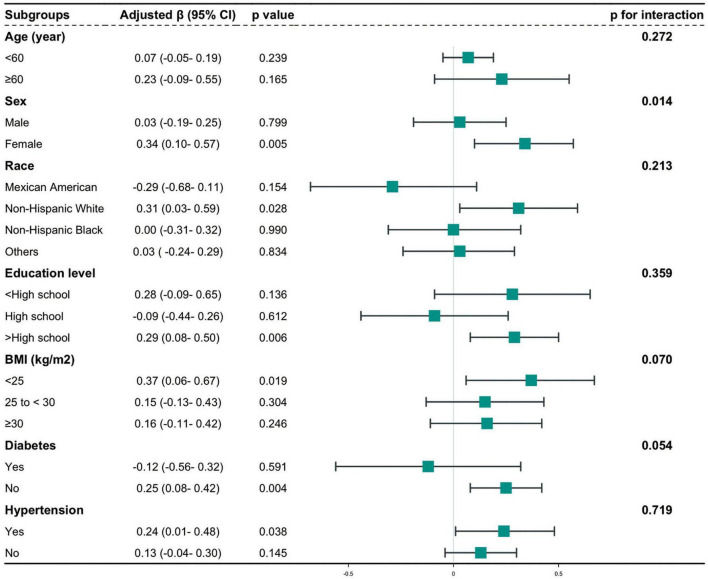
Hierarchical analysis and interaction analysis of the correlation between CMI and AAC scores. AAC, abdominal aortic calcification; CI, confidence interval; CMI, cardiometabolic index; BMI, body mass index.

**FIGURE 5:**
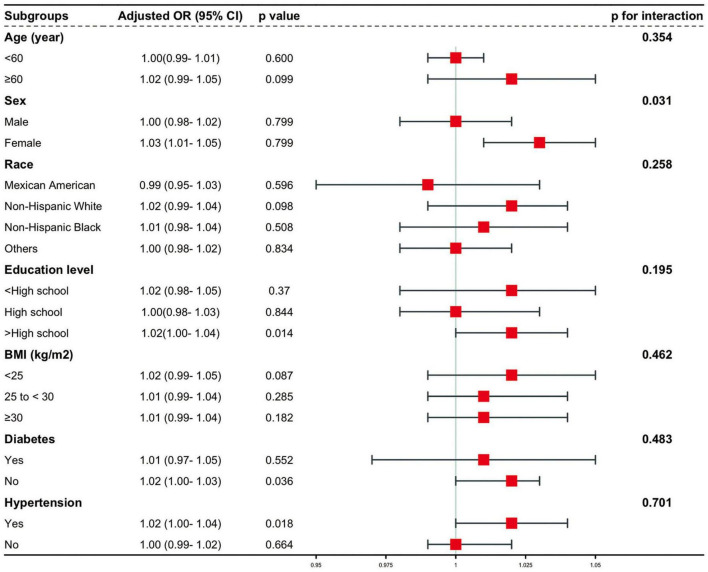
Stratified and interaction analysis of the association between CMI and the risk of severe AAC. AAC, abdominal aortic calcification; CMI, cardiometabolic index; CI, confidence interval; OR, odds ratio; BMI, body mass index.

To assess the stability of the relationship between CMI and AAC across various subgroups, a series of subgroup analyses were conducted. The interaction tests revealed no statistically significant differences in the correlation between lnCMI and AAC scores across the different subgroups (Figure 4 and Supplementary Table S2). These findings suggest that factors such as age, ethnicity, education level (below high school, high school, above high school), PIR, BMI, and diabetes status (present/absent) did not significantly affect this positive relationship (all interactions, *P* > 0.05). However, a notable interaction was observed in the sex subgroup (*P* < 0.05). The results of the subgroup analyses for lnCMI and severe AAC are shown in Figure 5 and Supplementary Table S3. In the age, race, education level, BMI, diabetes, and hypertension subgroups, the ORs and *P*-values did not show significant interaction differences. In contrast, within the sex subgroup, the β values for males and females differed significantly, indicating a meaningful interaction.

## Discussion

This cross-sectional analysis of 2,675 participants identified a statistically significant association between higher CMI levels and greater severity of AAC. This association remained evident after adjustment for potential confounders and was observed regardless of whether CMI was modeled as a continuous or categorical variable. Subgroup analyses demonstrated variation in the strength of this association across different populations, particularly among females, non-Hispanic White individuals, with higher educational attainment, those with hypertension, and those without diabetes. These findings indicate a statistical relationship between CMI and AAC burden in this cross-sectional dataset.

Since its introduction in 2015, the Cardiometabolic Index (CMI) has emerged as a composite indicator of visceral adiposity and dyslipidemia (42). Several studies have reported that CMI is associated with cardiovascular conditions and may offer advantages over traditional anthropometric indices in specific settings (43). The selection of CMI in our study was driven by its alignment with the pathophysiology of abdominal aortic calcification (AAC), which is influenced by both atherogenic lipid patterns and visceral fat accumulation. CMI captures these dual mechanisms by combining TG/HDL-C ratio with WHtR, a validated proxy for central obesity, both implicated in vascular aging processes. Compared to indices like LAP and VAI, CMI has been reported to show robust associations with metabolic dysfunction in large-scale studies. Cai et al. (44) reported that CMI outperformed VAI (AUC = 0.819 vs. 0.807) in identifying metabolic dysfunction-associated fatty liver disease (44). In a recent prospective cohort, Sun et al. (45) also found CMI to be more strongly correlated with acute pancreatitis incidence than LAP, suggesting its broader relevance to metabolic dysregulation (45). In contrast to LAP and VAI, which require sex-specific or population-specific coefficients and complex transformations, CMI is more practical and generalizable. WHtR, the central component of CMI, has outperforms BMI and WC in predicting cardiometabolic risk (46). CMI’s height-adjusted design also reduces stature-related confounding, making it more practical for routine screening in large population-based studies or clinical settings. LAP does not incorporate protective lipids such as HDL-C and is sex-specific, limiting generalizability. VAI includes regression-derived constants that may lack external validity across diverse populations. Moreover, visceral adiposity, reflected by WHtR, is associated with vascular calcification and atherosclerosis, supporting the relevance of CMI in AAC research (47). In addition, greater vulnerability of atherosclerotic lesions within the aorta was described for those with central adiposity (48). In light of these points, we believe that CMI represents a robust, generalizable, and physiologically integrative index, making it particularly suitable for investigating its association with vascular aging phenotypes such as AAC. Nonetheless, it is important to emphasize that our findings are based on cross-sectional data, and as such, CMI should not be interpreted as a causal or predictive tool for AAC.

The restricted cubic spline analysis was used to characterize the non-linear association between CMI and severe AAC in this cross-sectional study. The restricted cubic spline analysis revealed that when CMI exceeds approximately 0.66, the odds of severe abdominal aortic calcification increase progressively. This threshold may correspond to a level of cardiometabolic burden where central adiposity and atherogenic dyslipidemia are more strongly associated with vascular calcification. Although causality cannot be established due to the cross-sectional design, this inflection point may help characterize a population subgroup with elevated AAC burden in relation to metabolic risk. Higher CMI values were associated with greater diabetes prevalence, a finding consistent with prior observational studies. Hyperlipidemia is commonly observed in individuals with type 2 diabetes and is frequently associated with cardiovascular and renal complications in epidemiologic research (49). Interestingly, our study identified a numerically stronger association between CMI and AAC severity in non-diabetic individuals compared with diabetic ones. Research suggests that individuals with diabetes are more likely to use lipid-lowering medications, which often result in lower LDL cholesterol levels (50). The use of lipid-lowering therapy has been associated with reduced AAC burden in previous studies, which may help explain the weaker association between CMI and AAC observed in diabetic individuals in our analysis. Furthermore, our results indicate that elevated CMI levels in individuals with hypertension were more prominently associated with AAC severity, consistent with prior observational findings. Individuals with high blood pressure often exhibit metabolic and cardiovascular dysfunctions (51), which are frequently linked with metabolic abnormalities and vascular calcification (52, 53). Hypertension may modify the observed association between CMI and AAC, as the relationship appeared more pronounced among hypertensive individuals in our study. Chronic kidney disease (CKD) also commonly coexists with both diabetes and hypertension (54, 55). Individuals with CKD frequently exhibit lipid metabolism abnormalities (56), and previous studies have reported that lipid accumulation in the kidneys is associated with renal dysfunction, particularly in patients with glomerular disease or glycogen storage disorders (57). The scavenger receptor CD36 has been implicated in lipid metabolism dysregulation and may play a role in CKD pathophysiology, as suggested by previous experimental studies. The observed negative correlation between HDL-C and AAC severity is consistent with prior evidence suggesting a potential inverse relationship between HDL-C levels and vascular calcification (58). The OR for TGs and severe AAC was 1.07 (95% CI: 1.02–1.12, *P* = 0.014), indicating a positive correlation. This finding is consistent with earlier studies linking lipid profiles to cardiovascular health. For example, the LAP, which incorporates waist circumference and fasting triglycerides, has been shown in previous studies to be associated with increased cardiovascular risk (59). Although the limited AUC values suggest weak statistical separation, these findings remain relevant for understanding population-level metabolic associations. Given the multifactorial nature of AAC, which involves genetic, inflammatory, and metabolic pathways, it is unlikely that any single metabolic marker would achieve high discriminative accuracy on its own.

In our stratified analysis, a statistically significant interaction was observed between sex and CMI in relation to severe AAC, with women showing a stronger association than men. This may reflect a combination of biological and social factors possibly related to postmenopausal hormonal changes, altered lipid metabolism, and disparities in socioeconomic access to care among women. In vascular diseases, gender, socioeconomic status (SES), and ethnicity are crucial social determinants of health (SDHs) that contribute to health disparities and inequalities (60). Estrogen decline after menopause could disrupt lipid metabolism by increasing LDL-C and decreasing HDL-C levels, thereby promoting atherosclerotic processes, including vascular calcification (61). Moreover, postmenopausal women often exhibit increased central adiposity and atherogenic lipid profiles, both of which are captured by the CMI and are known contributors to vascular calcification (62). During post menopause, central adiposity has been associated with inflammatory and an increased risk of cardiovascular disease (63). Moreover, Depression is more commonly reported among women than men (64). This disparity is particularly pronounced during the menopausal transition. Fluctuating estrogen levels can disrupt the regulation of serotonin and norepinephrine, which may contribute to depression development (65). In postmenopausal women, depression has been associated with nearly a 50% increase in cardiovascular-related mortality (66). Socioeconomic factors also shape cardiometabolic risk profiles in sex-specific ways. In many lower-income countries, women tend to exhibit higher rates of obesity than men (67). Socioeconomic status (SES) in both childhood and adulthood influences the risk of metabolic syndrome (MetS) among women, with adult SES playing a particularly important role after menopause. Women from disadvantaged backgrounds face reduced access to healthcare and higher risks of underdiagnosed or unreported health conditions (68).

Individuals with lower socioeconomic status (SES) often exhibit higher rates of obesity, particularly in less urbanized regions. Lower educational attainment has been consistently associated with poorer dietary habits, reduced physical activity, and limited health literacy, all of which contribute to increased cardiometabolic risk (69). In contrast, those with higher education and income are more likely to adopt healthier lifestyles, seek timely medical care, and participate in comprehensive health plans (70). For instance, well-educated households are more responsive to dietary recommendations and more proactive in managing early symptoms and health conditions (71). Occupational stress and burnout have been associated with elevated cardiovascular risk in prior research (72). At the systemic level, economic disparities also influence healthcare access and quality. Regions with higher per capita GRP typically have better-funded hospitals, more advanced medical infrastructure, and greater access to skilled personnel, which together enhance service capacity. Conversely, economically disadvantaged communities face disproportionate burdens of disease and reduced access to preventive and specialized care (73).

The study found that CMI levels varied significantly by ethnicity, with higher values observed among non-Hispanic White individuals, Mexican Americans, and other groups. These differences may reflect underlying variations in body composition; for instance, Black individuals typically have higher lean mass and lower amounts of visceral and subcutaneous fat compared with Whites, which could contribute to their relatively lower CMI levels (74). While differences in BMI and poverty levels between Black and White men are less pronounced than among women, Black men consistently report lower alcohol consumption compared to White men (75). Stimulant medications, despite their clinical indications, have been disproportionately prescribed in affluent, predominantly White communities, raising concerns about overuse, and higher rates of obesity, alcohol dependence, and metabolic disorders in these populations (76). Ethnic background and experiences of marginalization have been associated with persistent health disparities, as socioeconomically advantaged populations tend to experience better health outcomes compared to underserved communities (77). Since 2005–2006, obesity rates among non-Hispanic Black men have remained stable, whereas Mexican American men have experienced a more rapid increase compared to non-Hispanic White men (78).

### Study strengths and limitations

This study conducted a cross-sectional analysis of data from American adults in the NHANES database to examine, for the first time, the association between the cardiometabolic index (CMI) and abdominal aortic calcification (AAC). Multivariable logistic regression and subgroup analyses were employed to explore this relationship and assess the consistency of findings across different population strata. These results provide descriptive epidemiologic insights that may serve as a foundation for future longitudinal studies on cardiometabolic markers and vascular calcification. However, Given the cross-sectional nature of NHANES, the associations observed in this study cannot establish causality. Therefore, all interpretations of our results are limited to correlations, rather than directional or predictive conclusions. Longitudinal studies are warranted to evaluate whether elevated CMI is temporally associated with AAC development or progression. Moreover, the statistical power of subgroup and interaction analyses in this study may be limited by sample size constraints within specific population subgroups. Future research should prioritize comprehensive cohort studies that track CMI patterns longitudinally and examine potential associations with changes in AAC burden over time. Additionally, further studies are needed to explore the biological pathways that may underlie the observed associations between CMI and vascular calcification. While CMI represents a composite indicator of cardiometabolic burden in population-level observational settings, it may not fully capture individual metabolic heterogeneity, particularly in genetically and environmentally diverse populations. Therefore, prospective data are warranted to better characterize the dynamic interplay between CMI components and cardiometabolic health. Furthermore, future studies should investigate whether integrating CMI with additional metabolic or imaging biomarkers may enhance its potential applicability in characterizing population-level cardiometabolic risk profiles in a non-invasive manner.

## Conclusion

This cross-sectional study provides preliminary evidence of a positive association between the cardiometabolic index (CMI) and the severity of abdominal aortic calcification (AAC) in a representative U.S. adult population. Although the AUC of 0.548 suggests modest association strength, higher CMI levels were statistically related to increased AAC severity. Given the cross-sectional design, no causal or temporal inferences can be made. These findings indicate that CMI may be associated with underlying cardiometabolic features that coexist with AAC burden in observational contexts.

## Data Availability

The datasets presented in this study can be found in online repositories. The names of the repository/repositories and accession number(s) can be found at: NHANES (http://www.cdc.go/nchs/nhanes.htm).
