# Supplementary material for: The cross-sectional association between cardiometabolic index and abdominal aortic calcification in U.S. adults: evidence from NHANES 2013–2014
Source: Front Nutr. 2025 Jul 2;12:1537795. doi: 10.3389/fnut.2025.1537795 (PMC12263387; doi:10.3389/fnut.2025.1537795)
Supplement: Supplementary file 1 [file Data_Sheet_1.zip › supplementary material/Table S1.docx]

|  |  | AAC Score | | Severe AAC | |
| --- | --- | --- | --- | --- | --- |
|  |  | β (95% CI) | p-value | OR (95% CI) | p-value |
|  | Continuous | 0.05 (-0.01- 0.12) | 0.117 | 1.07 (1.02- 1.12) | 0.014 |
|  | Categories |  |  |  |  |
|  | Q1 | 0 (Ref) |  | 1 (Ref) |  |
| TG | Q2 | 0.11 (-0.23- 0.44) | 0.539 | 1.54 (0.88- 2.69) | 0.151 |
|  | Q3 | 0.14 (-0.21- 0.49) | 0.425 | 1.33 (0.94- 1.87) | 0.130 |
|  | Q4 | 0.37 (0.00- 0.73) | 0.049 | 1.99 (1.28- 3.09) | 0.008 |
|  | P for tend | 0.11 (0.00- 0.23) | 0.056 | 1.21 (1.04- 1.42) | 0.027 |
|  |  |  |  |  |  |
|  | Continuous | -0.43 (-0.75- -0.11) | 0.008 | 0.58 (0.37- 0.91) | 0.031 |
|  | Categories |  |  |  |  |
|  | Q1 | 0 (Ref) |  | 1 (Ref) |  |
| HDL-C | Q2 | -0.40 (-0.73- -0.06) | 0.020 | 0.73 (0.40- 1.32) | 0.309 |
|  | Q3 | -0.38 (-0.74- -0.02) | 0.036 | 0.62 (0.38- 1.02) | 0.079 |
|  | Q4 | -0.57 (-0.95- -0.05) | 0.004 | 0.48 (0.24- 0.96) | 0.056 |
|  | P for tend | -0.17 (-0.29- -0.05) | 0.007 | 0.79 (0.65- 0.95) | 0.028 |
|  |  |  |  |  |  |
|  | Continuous | -2.57 (-5.27- 0.14) | 0.063 | 0.35 (0.02- 6.76) | 0.499 |
|  | Categories |  |  |  |  |
|  | Q1 | 0 (Ref) |  | 1 (Ref) |  |
| WHtR | Q2 | 0.57 (0.17- 0.98) | 0.006 | 1.90 (0.91- 3.96) | 0.108 |
|  | Q3 | 0.27 (-0.23- 0.78) | 0.292 | 1.23 (0.57- 2.64) | 0.602 |
|  | Q4 | 0.00 (-0.62- 0.62) | 1.000 | 0.96 (0.47- 1.92) | 0.900 |
|  | P for tend | -0.04 (-0.24- 0.16) | 0.708 | 0.92 (0.73- 1.15) | 0.463 |

Table S1. Associations between TG, HDL-C and WHtR and abdominal aortic calcification (AAC).

Adjusted for age, sex, race, and education level, smoking, alcohol use, BMI, PIR, DM, hypertension, CKD, CVD, asthma, arthritis, COPD, cancer, serum calcium, serum phosphorus, total 25-Hydroxyvitamin D, uric acid, and blood urea nitrogen based on Model 2. TG: triglyceride. HDL-C: high-density lipoprotein cholesterol. WHtR: waist-to-height ratio.
