# Supplementary material for: The cross-sectional association between cardiometabolic index and abdominal aortic calcification in U.S. adults: evidence from NHANES 2013–2014
Source: Front Nutr. 2025 Jul 2;12:1537795. doi: 10.3389/fnut.2025.1537795 (PMC12263387; doi:10.3389/fnut.2025.1537795)
Supplement: Supplementary file 1 [file Data_Sheet_1.zip › supplementary material/Table S2.docx]

**Table S2.** **Subgroup analysis of the association between lnCMI and AAC score.**

| **Subgroups** | **AAC score [β (95%CI)]** | **P-value** | **P for interaction** |
| --- | --- | --- | --- |
| **Age (year)** |  |  | 0.272 |
| < 60 years | 0.07 (-0.05- 0.19) | 0.239 |  |
| ≥ 60 years | 0.23 (-0.09- 0.55) | 0.165 |  |
| **Gender** |  |  | 0.014 |
| Male | 0.03 (-0.19- 0.25) | 0.799 |  |
| Female | 0.34 (0.10- 0.57) | 0.005 |  |
| **Race** |  |  | 0.213 |
| Mexican American | -0.29 (-0.68- 0.11) | 0.154 |  |
| Non-Hispanic White | 0.31 (0.03- 0.59) | 0.028 |  |
| Non-Hispanic Black | 0.00 (-0.31- 0.32) | 0.990 |  |
| Others | 0.03 ( -0.24- 0.29) | 0.834 |  |
| **Education level** |  |  | 0.359 |
| < High school | 0.28 (-0.09- 0.65) | 0.136 |  |
| High school | -0.09 (-0.44- 0.26) | 0.612 |  |
| > High school | 0.29 (0.08- 0.50) | 0.006 |  |
| **BMI (kg/m^2^)** |  |  | 0.070 |
| < 25 | 0.37 (0.06- 0.67) | 0.019 |  |
| 25 to < 30 | 0.15 (-0.13- 0.43) | 0.304 |  |
| ≥ 30 | 0.16 (-0.11- 0.42) | 0.246 |  |
| **Diabetes** |  |  | 0.054 |
| Yes | -0.12 (-0.56- 0.32) | 0.591 |  |
| No | 0.25 (0.08- 0.42) | 0.004 |  |
| **Hypertension** |  |  | 0.719 |
| Yes | 0.24 (0.01- 0.48) | 0.038 |  |
| No | 0.13 (-0.04- 0.30) | 0.145 |  |
