# Supplementary material for: The cross-sectional association between cardiometabolic index and abdominal aortic calcification in U.S. adults: evidence from NHANES 2013–2014
Source: Front Nutr. 2025 Jul 2;12:1537795. doi: 10.3389/fnut.2025.1537795 (PMC12263387; doi:10.3389/fnut.2025.1537795)
Supplement: Supplementary file 1 [file Data_Sheet_1.zip › supplementary material/Table S3.docx]

**Table S3 Subgroup analysis of the association between lnCMI and severe AAC.**

| **Subgroups** | **Severe AAC [OR (95%CI)]** | **P-value** | **P for interaction** |
| --- | --- | --- | --- |
| **Age (year)** |  |  | 0.354 |
| < 60 years | 1.00 (0.99- 1.01) | 0.600 |  |
| ≥ 60 years | 1.02 (0.99- 1.05) | 0.099 |  |
| **Gender** |  |  | 0.031 |
| Male | 1.00 (0.98- 1.02) | 0.799 |  |
| Female | 1.03 (1.01- 1.05) | 0.006 |  |
| **Race** |  |  | 0.258 |
| Mexican American | 0.99 (0.95- 1.03) | 0.596 |  |
| Non-Hispanic White | 1.02 (0.99- 1.04) | 0.098 |  |
| Non-Hispanic Black | 1.01 (0.98- 1.04) | 0.508 |  |
| Others | 1.00 (0.98- 1.02) | 0.834 |  |
| **Education level** |  |  | 0.195 |
| < High school | 1.02 (0.98- 1.05) | 0.370 |  |
| High school | 1.00 (0.98- 1.03) | 0.844 |  |
| > High school | 1.02 (1.00- 1.04) | 0.014 |  |
| **BMI (kg/m^2^)** |  |  | 0.462 |
| < 25 | 1.02 (0.99- 1.05) | 0.087 |  |
| 25 to < 30 | 1.01 (0.99- 1.04) | 0.285 |  |
| ≥ 30 | 1.01 (0.99- 1.04) | 0.182 |  |
| **Diabetes** |  |  | 0.483 |
| Yes | 1.01 (0.97- 1.05) | 0.552 |  |
| No | 1.02 (1.00- 1.03) | 0.036 |  |
| **Hypertension** |  |  | 0.701 |
| Yes | 1.02 (1.00- 1.04) | 0.018 |  |
| No | 1.00 (0.99- 1.02) | 0.664 |  |
